# COX-2 inhibition as a therapeutic strategy for bone loss in *Staphylococcus aureus* osteomyelitis

**DOI:** 10.1186/s10020-025-01202-9

**Published:** 2025-05-07

**Authors:** Yuhui Chen, Chao Li, Jishan Jia, Yuhui Jiang, Ping Zhang, Caiyu Cheng, Guangyan Zhang, Lang Gao, Xiang Yang, Jiawei Zhao, Kaiqun Li, Bin Yu

**Affiliations:** 1 Division of Orthopedics and Traumatology, Department of Orthopedics, Nanfang Hospital, Southern Medical University, No. 1838 North of Guangzhou Avenue, Guangzhou, 510515 Guangdong China; 2 Guangdong Provincial Key Laboratory of Bone and Cartilage Regenerative Medicine, Nanfang Hospital, Southern Medical University, No. 1838 North of Guangzhou Avenue, Guangzhou, 510515 Guangdong China; 3 Nanfang Hospital, Southern Medical University, No. 1838 North of Guangzhou Avenue, Guangzhou, 510515 Guangdong China

## Abstract

Bone loss in *Staphylococcus aureus* (*S. aureus*) osteomyelitis poses a serious challenge to orthopedic treatment, but the underlying mechanism of systemic osteoporosis caused by chronic infection is not completely clear. In this study, γ-irradiation-killed *S. aureus* (IKSA) was applied to simulate the inflammation and explore the mechanism of systemic bone loss caused by it. In this study, we found that the systemic application of IKSA caused bone loss in mice through increasing osteoclasts and decreasing osteoblasts. An immune response profile with up-regulated COX-2 is identified based on our transcriptional data from IKSA mice bone marrow cells. COX-2 expression is widely up-regulated in bone marrow immune cells, such as myeloid-derived suppressor cells (MDSCs), neutrophils and macrophages in the IKSA-treated mice. Mechanistically, COX-2 stimulated the increasing proportion of MDSCs and neutrophils and the inflammatory response of the bone marrow immune cells, that may regulate bone metabolism. Importantly, COX-2 inhibitor, celecoxib could rescue the bone loss induced by IKSA, which may reason from decrease of inflammatory gene expression in MDSCs, neutrophils and macrophages. Excitingly, COX-2 expression is also increased in bone marrow from mice and patients with *S. aureus* osteomyelitis. These findings suggested a therapeutic potential for inhibiting COX-2 in combating bone loss in *S. aureus* osteomyelitis.

**Supplementary Information:**

The online version contains supplementary material available at 10.1186/s10020-025-01202-9.

## Background

Osteomyelitis is a chronic bone tissue infection characterized by significant inflammation and ongoing bone destruction (Lew and Waldvogel 2004). As the predominant pathogen, *Staphylococcus aureus* (*S. aureus*) is responsible for approximately 80% of clinical cases (Berendt and Byren 2004). *S. aureus* infection not only leads to localized inflammatory bone destruction but also exerts systemic skeletal effects (Masters et al. 2022). Importantly, osteomyelitis is associated with an increased risk of fragility fractures occurring at locations distant from the infection site (Hsieh et al. 2019). While the mechanisms through which osteomyelitis induces systemic bone loss remain incompletely understood, chronic inflammation associated with this condition is characterized by elevated levels of circulating inflammatory cytokines, such as prostaglandin E2 (PGE2), interleukin-1, interleukin-6 and tumor necrosis factor-alpha. These cytokines may contribute to systemic bone loss, leading to a decrease in bone strength (Jones et al. 2011; Granata et al. 2022).

In our research, a model of systemic chronic inflammation was developed by using γ-irradiation-killed *S. aureus* (IKSA). This approach employs γ-irradiation to effectively kill bacteria while minimizing alterations to the bacterial cell structure. As a result, the integrity of the bacterial cell envelope is largely preserved, maintaining the tumor necrosis factor-alpha inducing activity caused by their lipopolysaccharides and lipid composition, unlike traditional antibiotic or heat-based bacterial killing methods (Correa et al. 2019). This methodology allows for the simulation of persistent inflammation while reducing the risk of additional infections.

Selective inhibitors of cyclooxygenase-2 (COX-2) are widely used pharmacological treatments for musculoskeletal pain in osteomyelitis patients (Hsu et al. 2019). A multicenter study indicated that reduced bone mineral density (BMD) is attributed to COX-2-selective inhibitors in men (O’Connor and Lysz 2008); in contrast, these inhibitors appear to increase BMD after menopause in women, highlighting the different roles of COX-2 in bone regulation (Salari and Abdollahi 2009). COX-2 is crucial for prostaglandin synthesis and is actively involved in both inflammatory responses and the processes of bone formation and repair (Zhang et al. 2002; Robertson et al. 2006). Nonsteroidal anti-inflammatory drugs (NSAIDs) exert their biological effects, at least in part, by inhibiting COX-2 and reducing PGE2 production (Arfeen et al. 2024), which subsequently promotes the differentiation and secretion of inflammatory cytokines by immunoregulatory cells, including myeloid-derived suppressor cells (MDSCs) (Fujita et al. 2011), neutrophils (Carvalho et al. 2022), and macrophages (Kulesza et al. 2023). However, the involvement of COX-2 in systemic bone loss linked to *S. aureus* osteomyelitis is not fully understood.

Our research demonstrated that IKSA significantly triggered bone loss in mice by suppressing bone formation and enhancing bone resorption. Moreover, COX-2 levels were elevated in the bone marrow of mice intraperitoneally injected with IKSA, as well as in mice with *S. aureus* hematogenous osteomyelitis and individuals with *S. aureus* osteomyelitis. Additionally, our findings indicated that the administration of celecoxib, which inhibits COX-2, effectively mitigated bone loss in vivo. These results suggest that targeting COX-2 may be an effective strategy to counteract the bone loss associated with *S. aureus* infection.

## Methods

### Patients and specimens

All participants were selected from the inpatients in the Department of Orthopedics and Traumatology of Nanfang Hospital. Among the 7 participants in Osteomyelitis group, the median age was 55 years, with 72% male and 28% female. Participants were diagnosed with S. aureus osteomyelitis based on clinical presentations and microbiological culture, following lower limb fracture fixation. Similarly, in the control group, the median age of 7 participants was 54 years, with 72% male and 28% female participants. Control participants had closed fractures in the femur or tibia, without infection symptoms or positive results of bacterial culture. The research was ethically sanctioned by the Ethics Committee at Nanfang Hospital. Osteomyelitis diagnosis followed the guidelines set by Lew and Waldvogel. Inclusion criteria: adults aged 18–65 years; male or female; hospitalization due to lower limb or femoral head fracture. Exclusion criteria: presence of severe systemic diseases such as uncontrolled diabetes mellitus, cardiovascular diseases, or immunocompromised states; active infections other than osteomyelitis; participants diagnosed with tumors. Each participant in this research provided informed consent before taking part. The gathered samples were quickly enclosed and conveyed to the laboratory in less than 1 h. For Western blot analysis, the bone marrow encircling the infection area or intramedullary reaming slot and the fracture area were collected and preserved at −80 °C. For immunohistochemical staining, the surrounding bone marrow was decalcified and subsequently embedded in paraffin following fixation in 4% paraformaldehyde. (Approval Number: NFEC-2020-074).

### Animals and experimental protocol

All procedures involving animals received approval from the Institutional Animal Care and Use Committee of Nanfang Hospital. Ten-week-old male C57BL/6 mice were utilized to create the animal models (Approval Number: NFYY-2022-01037). The decision to utilize male mice was primarily based on the need to minimize hormonal fluctuations that can influence experimental outcomes. Female mice experience cyclical hormonal changes, particularly during the estrous cycle, which can introduce variability in physiological responses and complicate data interpretation (Zanotti et al. 2014; Osipov et al. 2022). By improving the previous research (Deng et al. 2023), The IKSA group mice were treated with 1 × 10^8^ CFUs of IKSA by intraperitoneal injection every 3 days, whereas the control group mice were administered an equivalent volume of PBS. The treatment lasted for 6 weeks. Celecoxib (MedChemExpres, HY-14398) was evenly mixed in 1% methyl cellulose (Acros Organics, A0342031). The mice in the celecoxib-treated group were intragastrically administered a 10 mg/kg celecoxib suspension, and the vehicle-treated group was intragastrically administered methyl cellulose with equal volume. According to previous research (Ji et al. 2020), the *S. aureus* hematogenous osteomyelitis mice were treated with 1 × 10^5^ CFU of *S. aureus* via intravenous injection.

### Bacterial strains and preparation of IKSA

*S. aureus* strains were obtained from the Laboratory Medicine Department of Nanfang Hospital. A single bacterial colony was transferred to tryptic soy broth (TSB) and incubated at 37 °C for 16–18 h under constant agitation at 200 rpm. The concentration of *S. aureus* was set as 5 × 10^8^ CFUs/mL with PBS after the bacteria were washed twice. *S. aureus* was subjected to 1000 Gy γ-irradiation with a Cobalt-60 γ source on ice (Jones et al. 2011), and dead bacteria were subsequently stored at −80 °C.

### Microcomputed tomography analysis

For microcomputed tomography analysis, high-resolution micro-CT (Skyscan 1276) was used to scan the femurs obtained from the mice at a 6.5 μm resolution after fixation in 4% paraformaldehyde for 24 h. For bone analysis, the 0.442–1.642 mm distal femur from the growth plate was set as the region of interest. The images were reconstructed with CTvol (version 2.2.3.0) and analyzed via a CT Analyzer (version 1.15.4.0). The main parameters included the trabecular bone volume/tissue volume (BV/TV), trabecular bone number (Tb. N), trabecular bone thickness (Tb. Th), trabecular bone separation (Tb. Sp), trabecular bone pattern factor (Tb. Pf), cortical bone crossectional thickness (Ct. Th), cortical bone crossectional area (Ct. Ar).

### Histology and immunohistochemical staining

Mouse femurs were sectioned at 4 μm with a microtome (Leica RM2245). A hematoxylin and eosin (H&E) staining procedure was used for structural analysis. For IHC staining, bone sections were subjected to antigen retrieval for 40 min at 70 °C and incubated with 10% goat serum for 1 h at ambient temperature to block nonspecific binding after endogenous peroxidases were quenched. Following incubation with primary antibodies against COX-2 (Abcam, ab179800) and osteocalcin (Affinity, DF12303) overnight at 4 °C, the sections were incubated with an HRP-conjugated secondary antibody (HuaBio, HA1001) for 1 h at room temperature. A DAB Kit (ZSGB-Bio, ZLI-9017) was used to visualize the immunohistochemical staining. Tartrate-resistant acid phosphatase (TRAP) staining of tissue sections was performed via a TRAP kit (FUJIFILM Wako, 294–67001). A fluorescence microscope (Olympus, BX63) was used to capture the images.

### RNA-seq analysis

Total RNA from the femoral bone marrow cells of the mice was extracted via RNAex Pro Reagent (Accurate Biology, 21102), and sequencing analysis was performed on a NovaSeq 6000 (Illumina). The expression of transcripts was calculated via the counts per million reads (CPM) method. Differential expression analysis was conducted with the DESeq2 tool. Enrichment analysis for Gene Ontology (GO) terms was conducted via the clusterProfiler package (version 4.10.0). Additionally, gene set enrichment analysis (GSEA) was executed with GSEA software (version 4.3.3), and the resulting data were visualized through the R package ggplot2 (version 3.4.4).

### Flow cytometry

The femurs and tibias of the mice were flushed to obtain bone marrow cells via a 25-gauge needle, and the cells were filtered through a 70 μm tissue sieve. Bone marrow cells were incubated with an anti-CD16/32 antibody for 10 min and then stained with antibodies specific for BV421-CD11b (LM2, Biolegend, 393113), FITC-Gr-1 (RB6-8 C5, Biolegend, 108405), PE-Cy7-Ly6G (1 A8, Biolegend, 127671), APC-Cy7-F4/80 (BM8, Biolegend, 123117), APC-CD11b (M1/70, Invitrogen, 17–0112-81), PerCP-Cy5.5-Gr-1 (RB6-8 C5, Invitrogen, 45–5931-80), FITC-Ly6G (1 A8, Invitrogen, 11–9668-80), and eFluor450-F4/80 (BM8, eBioscience, 48–4801-82) for 30 min on ice. The cells were fixed for 5 min with 10% formalin, washed, and then incubated for 30 min in Fix Perm solution (BioLegend, 426803). The cells were washed and then stained for 30 min with an anti-COX-2 antibody (Abcam, ab179800). After washing, PE-Goat anti mouse Secondary Antibody (eBioscience, 12–4010-82) was incubated. And then. The cells were subsequently washed and analyzed via the CytoFLEX system (Beckman Coulter).

### Primary cell culture

To derive MDSCs, as previously described (Choi et al. 2022), freshly isolated bone marrow cells from femurs and tibias were cultured in RPMI 1640 medium enriched with 10% fetal bovine serum (FBS, Excell, 16000-044), 40 ng/mL murine GM‒CSF (Sinobiological, 51048-MNAH), 40 ng/mL murine IL‒6 (Sinobiological, 50136-MNAE) and 1% penicillin‒streptomycin solution (Gibco, 15140122) for 4 days. For neutrophil preparation, as previously described (Yu et al. 2022), neutrophils derived from bone marrow were isolated via centrifugation with a Percoll (GE Healthcare) discontinuous gradient (75%, 66%, and 52%), and the cells were collected from the interface between the 75% and 66% layers. Bone marrow cells were harvested freshly from femurs and tibias to generate bone marrow-derived macrophages (BMDMs). The cells were cultured for 7 days in RPMI 1640 medium containing 10% FBS, 30% L929 supernatant, and 1% penicillin‒streptomycin solution.

### RNA isolation and quantitative RT‒PCR

Total RNA from cells and tissues was extracted via AG RNAex Pro Reagent (Accurate Biology, 21102). An Evo M-MLV RT Kit (Accurate Biology, AG11711) was used to carry out reverse transcription. Real-time quantitative PCR (qPCR) was performed on a QuantStudio 5 Real-Time PCR System (Thermo Scientific). The Supplementary Table lists the PCR primers and experimental conditions. The expression levels of the target genes were quantified via the 2^−ΔΔCT^ method, with *Gapdh* serving as the reference gene for normalization.

### Western blotting

Proteins were extracted from cells and tissues via RIPA lysis buffer (Epizyme Biotech, PC101) supplemented with a cocktail of protease and phosphatase inhibitors. (MedChemExpress, HY-K0013). The protein concentrations were quantified via a BCA Kit (Epizyme Biotech, ZJ101). The proteins were initially resolved via SDS‒PAGE and subsequently transferred to PVDF membranes. After transfer, the membranes were blocked. The PVDF membranes were incubated with primary antibodies against COX-2 (Abcam, ab179800) and GAPDH (Abcam, ab8245) overnight at 4 °C. The next day, the membranes were exposed to a secondary antibody and incubated at ambient temperature for 1 h. The signals were developed via enhanced chemiluminescence (ECL) (NCM Biotech, P10200) and detected via autoradiography via a chemiluminescence apparatus (BLT).

### Statistical analysis

Each experiment was performed with a minimum of three repetitions. The results are expressed as the mean values accompanied by their standard errors. Statistical evaluation was carried out via GraphPad Prism, version 9.5, software. Unpaired Student’s *t* tests were used for two-group comparisons. For multiple group analyses, one-way ANOVA with Tukey’s post hoc test was applied. A *p* value less than 0.05 was considered to indicate statistical significance.

## Results

### IKSA caused bone loss in mice through the inhibition of bone formation and stimulation of bone resorption

**Fig. 1:**
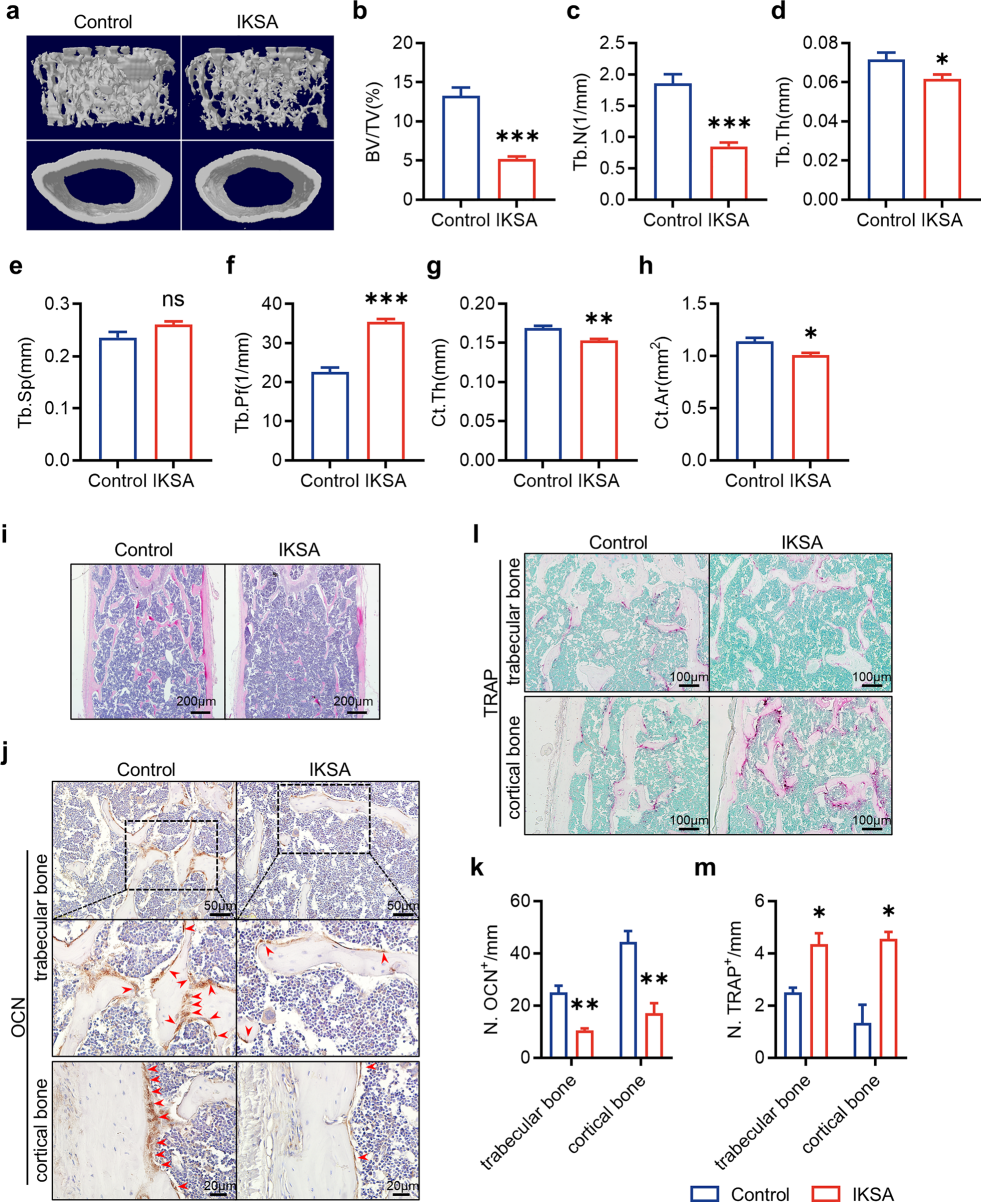
IKSA induced bone loss by suppressing bone formation and activating bone resorption in mice. Micro-CT reconstruction images showcased both trabecular and cortical bone structures (**a**), quantitative evaluation was conducted on the trabecular bone fraction (BV/TV) (**b**), number of trabeculae (Tb. N) (**c**), thickness of trabeculae (Tb. Th) (**d**), separation between trabeculae (**e**), trabecular bone pattern factor (Tb. Pf) (**f**), cortical bone thickness (Ct. Th) (**g**) and cortical bone crosssectional area (Ct. Ar) (**h**) of the femurs from IKSA-treated and control mice. *n* = 6/group, * *p* < 0.05, ** *p* < 0.01, *** *p* < 0.001. **i** Typical H&E-stained femoral sections from IKSA-treated and control mice are shown. Scale bars, 200 μm. Typical images (**j**) and quantitative analysis (**k**) of OCN immunohistochemical staining on the trabecular bone and cortical bone of femurs from IKSA-treated and control mice. *n* = 3/group, ** *p* < 0.01. Scale bars, 50 μm. Red arrows indicate OCN^+^ cells. Representative TRAP staining images (**l**) and quantification (**m**) of the trabecular bone and cortical bone in femurs of IKSA-treated and control mice. Scale bars, 100 μm. *n* = 3/group, * *p* < 0.05

To investigate how IKSA influences bone metabolism, we employed micro-CT imaging to examine mouse bone tissue. As depicted in Fig. 1a, administering IKSA led to marked bone loss after a 6-week period. Quantitative analyses of structural parameters in the distal femur clearly demonstrated these alterations. Specifically, there was a marked reduction in the BV/TV (Fig. 1b). This decrease in the BV/TV was correlated with a marked reduction in the Tb. N, a moderate decrease in the Tb. Th, and an increase in the Tb. Pf (Fig. 1c–f). Moreover, Ct. Th and Ct. Ar was significantly reduced in the IKSA group (Fig. 1g, h).

H&E staining revealed a significant reduction in both the Tb. N and Tb. Th of IKSA-treated mouse femurs (Fig. 1i). Given that the observed bone loss arises from an imbalance between osteogenesis and osteoclast activity, we further examined markers related to these processes. Immunohistochemical staining for osteocalcin (OCN) revealed that osteoblastic activity in the IKSA group was significantly lower than that in the control group, whether in trabecular bone or cortical bone (Fig. 1j, k; Fig. S2a, b). Furthermore, TRAP staining revealed a significant increase in TRAP^+^ cell counts along the trabecular bone and cortical bone surface in IKSA-treated mice compared with control mice (Fig. 1l, m; Fig. S2c, d). In summary, these results indicate that IKSA impairs bone formation through the suppression of osteogenesis and enhances bone resorption by stimulating osteoclastogenesis.

### Transcriptome analysis of bone loss induced by IKSA

**Fig. 2:**
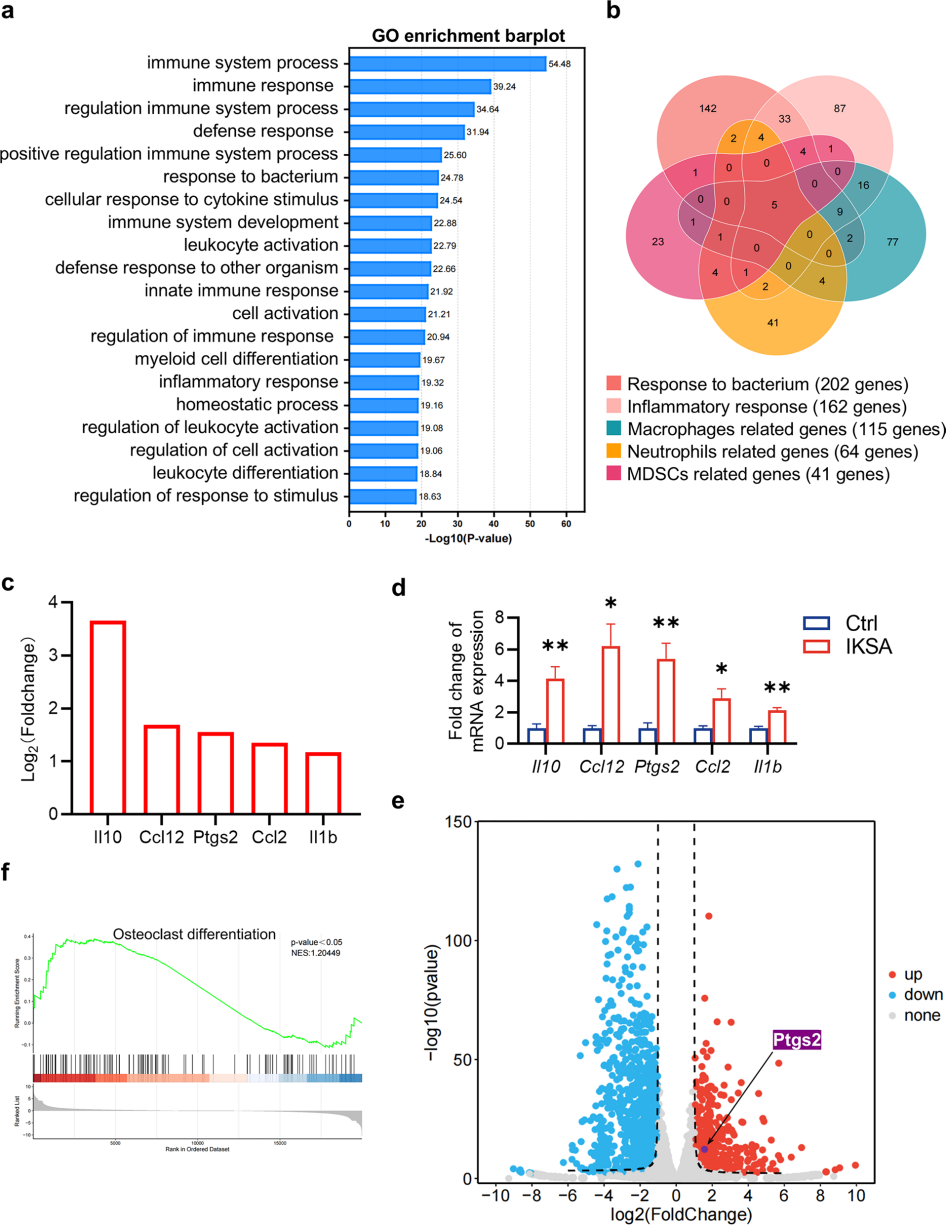
Transcriptome analysis of bone loss induced by IKSA. **a** Gene Ontology (GO) analysis of DEGs in bone marrow cells from mice that were administered IKSA intraperitoneally for 6 weeks. *n* = 4/group. GO enrichment analysis was performed with the help of the clusterProfiler package in R, with *p* value adjustments applied via the Benjamini‒Hochberg method. GO categories with an adjusted *p* value less than 0.05 were considered significantly enriched. **b** Venn diagram illustrating DEGs involved in the response to bacterium and the inflammatory response among macrophages, neutrophils and MDSCs. **c** A subset of inflammatory molecules notably increased in the femurs of mice treated with IKSA. **d** Quantitative analysis of the mRNA expression of *Il10*, *Ccl12*, *Ptgs2*, *Ccl2* and *Il1b* in mice treated with IKSA. *n* = 4/group. **e** Volcano plot of genes upregulated in the femurs of mice treated with IKSA. **f** GSEA of the “osteoclast differentiation” gene module among the DEGs

To characterize the molecular responses to IKSA in mice, we conducted transcriptome analysis of femurs from both the IKSA and control groups at 6 weeks post-injection via high-throughput sequencing. GO enrichment analysis of the DEGs revealed that genes associated with immune system function, response, and regulation presented increased expression (Fig. 2a). Our subsequent analysis focused on DEGs. Related to the bacterial response and inflammation in immune cells (macrophages, neutrophils and MDSCs), revealing 5 genes that are commonly modulated: *IL10*, *Ccl12*, *Ptgs2* (COX-2), *Ccl2* and *IL1b* (Fig. 2b, c). qPCR analysis demonstrated that mRNA expression of *IL10*, *Ccl12*, *Ptgs2*, *Ccl2* and *IL1b* was significantly elevated in IKSA femurs relative to control femurs (Fig. 2d). Notably, *Ptgs2* was significantly upregulated in the IKSA group (Fig. 2e). Additionally, GSEA revealed that IKSA treatment was primarily positively correlated with osteoclast differentiation. The expression of *Ptgs2* might contribute to the regulation of bone metabolism under IKSA treatment conditions (Fig. 2f).

### COX-2 expression in immune cells from the bone marrow of IKSA mice was upregulated

**Fig. 3:**
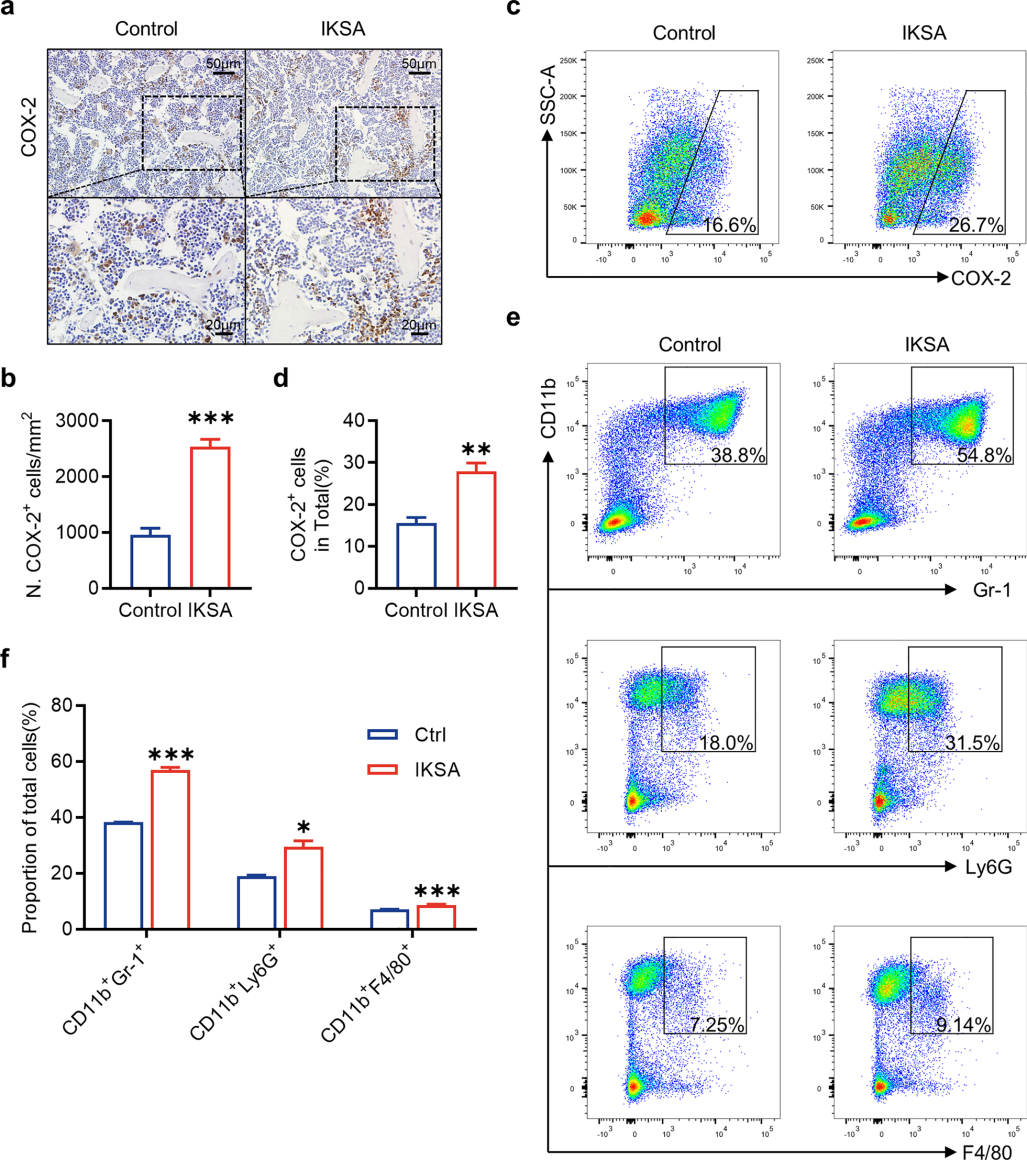
The expression of COX-2 was upregulated, and the proportions of immune cells in the bone marrow of IKSA-treated mice were increased. Typical images (**a**) and quantitative analysis (**b**) of COX-2 immunohistochemical staining in the femurs of control and IKSA-treated mice. Scale bars, 50 μm. *n* = 3/group, *** *p* < 0.001. Typical images (**c**) of flow cytometry and quantitative analysis (**d**) of COX-2^+^ cell proportions in the bone marrow of IKSA-treated and control mice. *n* = 4/group, ** *p* < 0.01. Typical images (**e**) of flow cytometry and quantitative analysis (**f**) of the proportions of MDSCs (CD11b^+^Gr-1^+^), neutrophils (CD11b^+^Ly6G^+^) and macrophages (CD11b^+^F4/80.^+^) in the bone marrow of control and IKSA-treated mice. *n* = 4/group, * *p* < 0.05, *** *p* < 0.001

**Fig. 4:**
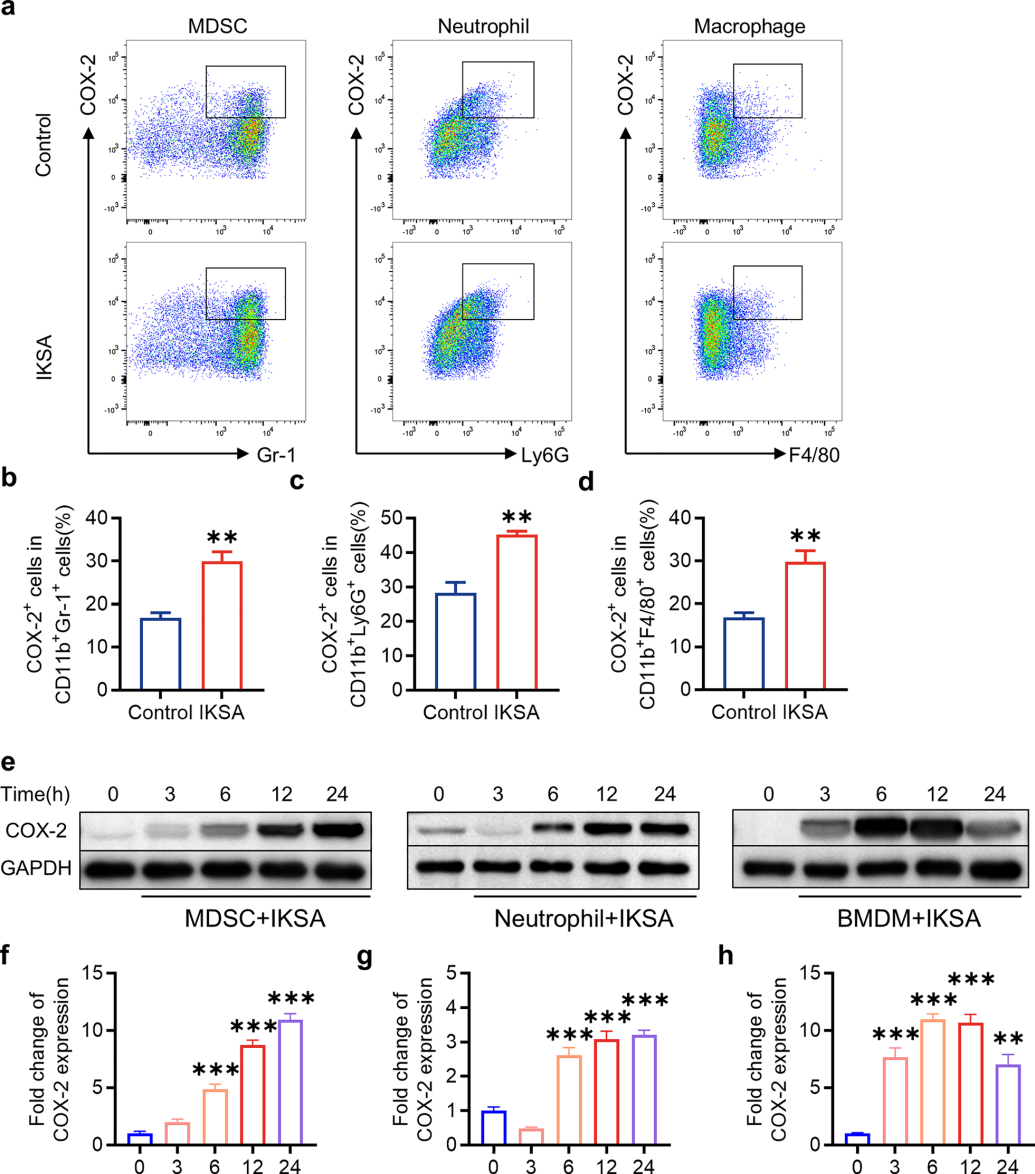
COX-2 expression in immune cells was upregulated by IKSA in vivo and in vitro. Typical flow cytometry images (**a**) and quantitative analysis of the proportions of COX-2.^+^ cells among MDSCs (**b**), neutrophils (**c**) and macrophages (**d**) from the bone marrow of IKSA-treated and control mice. *n* = 4/group, ** *p* < 0.01. Typical images of western blots (**e**) and quantitative analysis of the protein levels of COX-2 in MDSCs (**f**), neutrophils (**g**) and BMDMs (**h**) infected with IKSA (MOI = 10) for various durations (3, 6, 12 and 24 h). *n* = 3/group, ** *p* < 0.01, *** *p* < 0.001

To investigate the association between COX-2 and bone metabolism in the context of IKSA, we performed immunohistochemical staining and flow cytometry to analyze COX-2 expression in mouse bone marrow at 6 weeks post infection. These findings indicated a marked increase in COX-2 expression in bone marrow cells derived from the IKSA group (Fig. 3a–d). Recent evidence suggests that immune cells (neutrophils, macrophages and MDSCs) serve as key factors influencing bone metabolism during inflammatory states (Kirkwood et al. 2018; Cai et al. 2021; Schlundt et al. 2021). Using flow cytometry, we analyzed alterations in the quantity of these immune cells present in the bone marrow of mice subjected to IKSA treatment. Our observations revealed a significant increase in the proportion of CD11b^+^Gr-1^+^, CD11b^+^Ly6G^+^ and CD11b^+^F4/80^+^ cells in the femurs of IKSA-treated mice compared with those of control mice (Fig. 3e, f). Notably, the expression of COX-2 in these three immune cell populations was also elevated in the femurs of IKSA-treated mice (Fig. 4a–d).

To further investigate the temporal effects of IKSA infection on COX-2 expression, we subjected MDSCs, BMDMs, and neutrophils to IKSA at a multiplicity of infection (MOI) of 10. Our findings demonstrated a notable elevation in COX-2 levels across all three types of immune cell populations following IKSA infection (Fig. 4e–h). In summary, these results indicate that the activation of COX-2 expression is likely driven by *S. aureus* infection itself rather than by secreted virulence factors.

### The bone loss induced by IKSA in mice was relieved by a COX2-selective inhibitor celecoxib

**Fig. 5:**
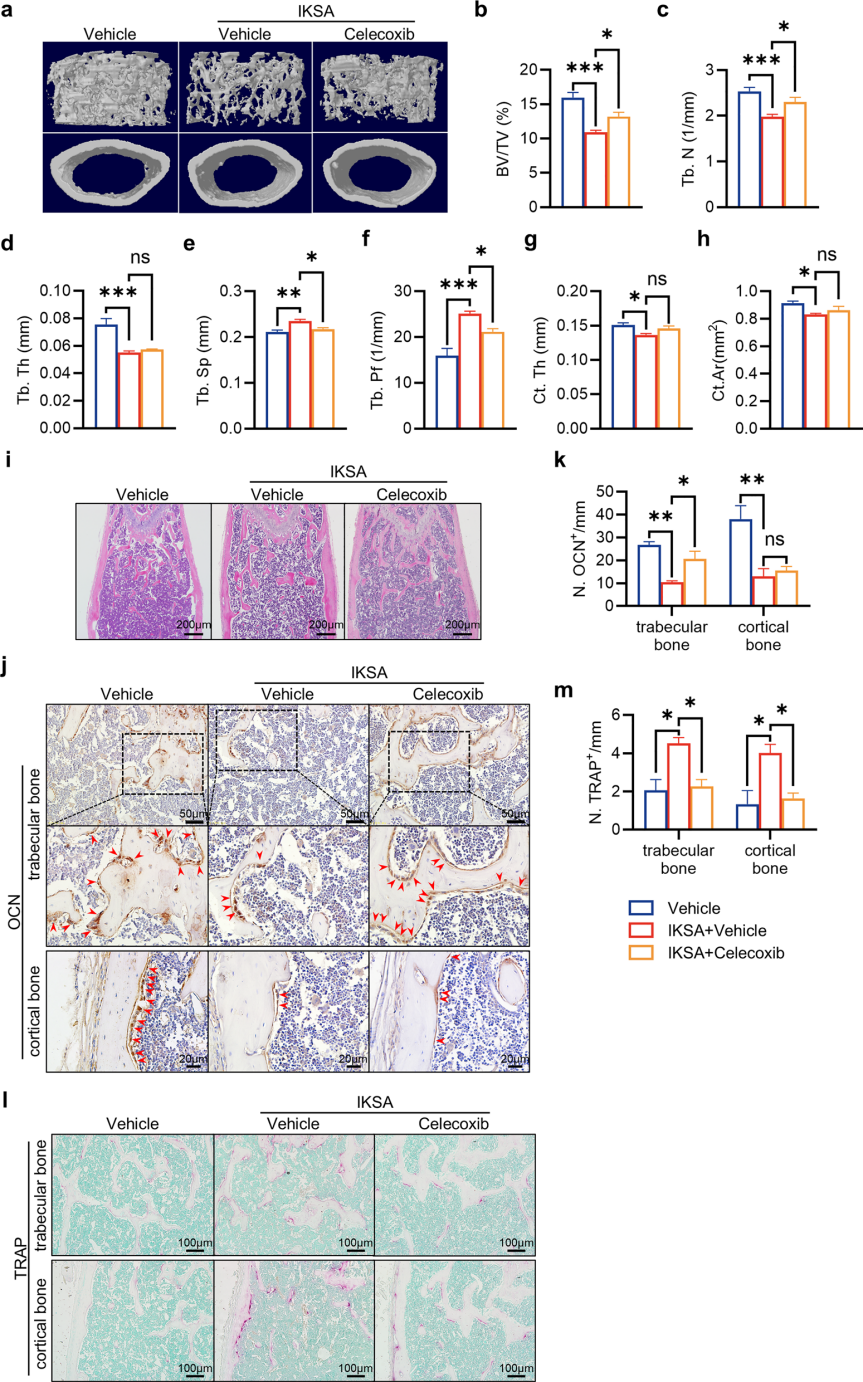
The bone loss induced by IKSA can be relieved by the COX-2 selective inhibitor celecoxib in mice. Micro-CT reconstruction images showcasing both trabecular and cortical bone structures (**a**), quantitative analysis of BV/TV (**b**), Tb. N (**c**), Tb. Th (**d**), Tb. Sp (**e**), Tb. Pf (**f**), Ct. Th (**g**) and Ct. Ar (**h**) of femurs from vehicle control mice and IKSA-treated mice with intragastric administration of vehicle or celecoxib. *n* = 5/group, * *p* < 0.05, ** *p* < 0.01. **i** Typical H&E-stained femoral sections from vehicle control mice and IKSA-treated mice with intragastric administration of vehicle or celecoxib. Scale bars, 200 μm. Typical images (**j**) and quantitative analysis (**k**) of OCN immunohistochemical staining on the trabecular bone and cortical bone of femurs from vehicle control mice and IKSA-treated mice with intragastric administration of vehicle or celecoxib. *n* = 3/group, * *p* < 0.05. Red arrows indicate OCN^+^ cells. Scale bars, 50 μm. Typical TRAP staining images (**l**) and quantitative analysis (**m**) of the trabecular bone and cortical bone in femurs of vehicle control mice and IKSA-treated mice with intragastric administration of vehicle or celecoxib. Scale bars, 100 μm. *n* = 3/group, ** *p* < 0.01

To determine whether inhibiting COX-2 signaling prevents the bone loss associated with *S. aureus* osteomyelitis, we treated IKSA-infected mice with the selective COX-2 inhibitor celecoxib. Surprisingly, blocking COX-2 signaling effectively rescued bone loss. The structure of the femoral bones was assessed via micro-CT imaging, which revealed that Vehicle and IKSA + Vehicle groups showed the same changes as Fig. 1, and IKSA + celecoxib-treated mice presented a notable increase in trabecular bone mass relative to that in the IKSA + Vehicle group. This increase was accompanied by increases in BV/TV and Tb. N and a decrease in Tb. Sp and Tb. Pf (Fig. 5a–h). Furthermore, H&E staining demonstrated a notable reduction in bone loss in celecoxib-treated mice. (Fig. 5i). Additionally, mice treated with IKSA + celecoxib presented an increase in OCN^+^ cells and a decrease in TRAP^+^ cells on the trabecular bone surface, in contrast to those treated with IKSA + Vehicle. Interestingly, in the endocortical region, although the number of osteoblasts has not changed significantly, the number of osteoclasts has decreased significantly in IKSA + celecoxib group. The change did not rescue the reduction of cortical bone in the end, likely due to osteogenic function has not been fully improved. (Fig. 5j–m; Fig. S3a–d). In summary, these findings suggest that the inhibition of COX-2 effectively reduces trabecular bone loss in IKSA mice.

### Celecoxib regulated the number and inflammatory response of immune cells in IKSA mice bone marrow

**Fig. 6:**
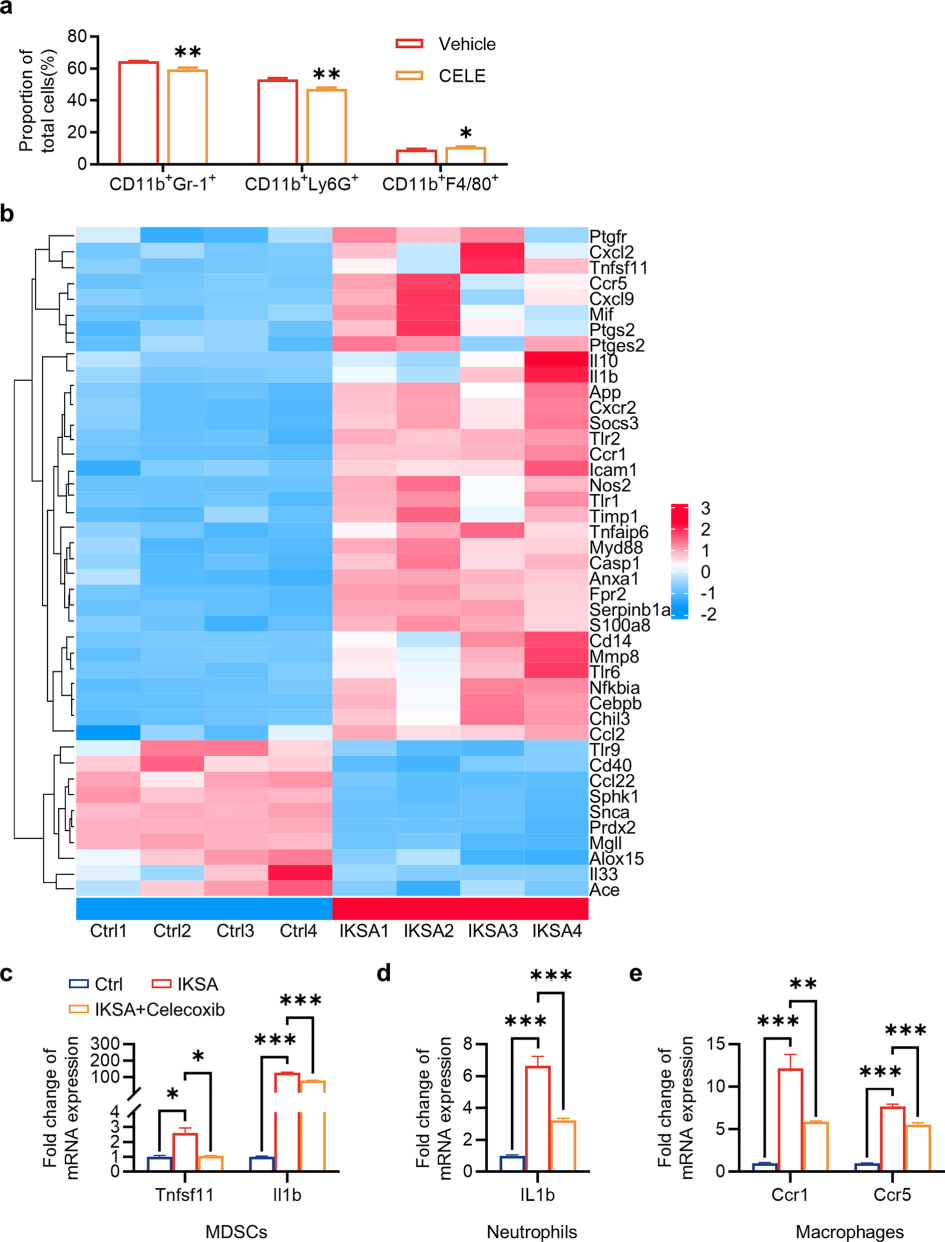
Celecoxib reduced the proportion of MDSCs and neutrophils and impaired osteoclast function through decreasing the expression of some genes associated with inflammation. **a** Quantitative analysis of the proportions of MDSCs, neutrophils and macrophages in the bone marrow of IKSA-treated mice treated intragastrically with vehicle or celecoxib. *n* = 4/group, * *p* < 0.05, ** *p* < 0.01. **b** Heatmap of 43 DEGs associated with COX-2 in the STRING database. **c** Quantitative analysis of *Tnfsf11* and *Il1b* mRNA expression in MDSCs treated with IKSA (MOI = 10) or IKSA + celecoxib for 12 h. *n* = 3/group, * *p* < 0.05, *** *p* < 0.001. **d** Quantitative analysis of *Il1b* mRNA expression in neutrophils treated with IKSA (MOI = 10) or IKSA + celecoxib for 12 h. *n* = 3/group, *** *p* < 0.001. **e** Quantitative analysis of the mRNA expression of *Ccr1* and *Ccr5* in macrophages treated with IKSA (MOI = 10) or IKSA + celecoxib for 12 h. *n* = 3/group, ** *p* < 0.01, *** *p* < 0.001

To explore the functional role of COX-2 in immune cells within the bone marrow of IKSA-infected mice, we treated the mice with celecoxib. We found that celecoxib significantly suppressed the expansion of MDSCs and neutrophils induced by IKSA while increasing the proportion of macrophages (Fig. 6a). Additionally, we performed protein‒protein interaction (PPI) analysis between COX-2 and DEGs associated with the inflammatory response via the STRING database, which identified 43 genes that interact with COX-2, as shown in the heatmap (Fig. 6b). Next, we tested for changes in the expression of these genes in immune cells (MDSCs, neutrophils, and macrophages) stimulated with IKSA in vitro and assessed whether celecoxib could reverse these changes. In MDSCs, the gene expression of *Tnfsf11* (RANKL) and *IL-1b* was elevated by IKSA, and celecoxib reversed these changes (Fig. 6c). In neutrophils, *IL-1b* expression was also upregulated by IKSA, and celecoxib effectively reversed this change (Fig. 6d). In macrophages, the gene expression of *Ccr1*, *Ccr5*, *Cxcl2* and *IL-1b* was increased by IKSA, and celecoxib reversed the changes in *Ccr1* and *Ccr5* (Fig. 6e). Collectively, these results suggest that celecoxib can regulate both the number and inflammatory response of immune cells in IKSA mice bone marrow.

### COX-2 expression in the bone marrow is upregulated in patients and mice with *S. aureus* osteomyelitis

**Fig. 7:**
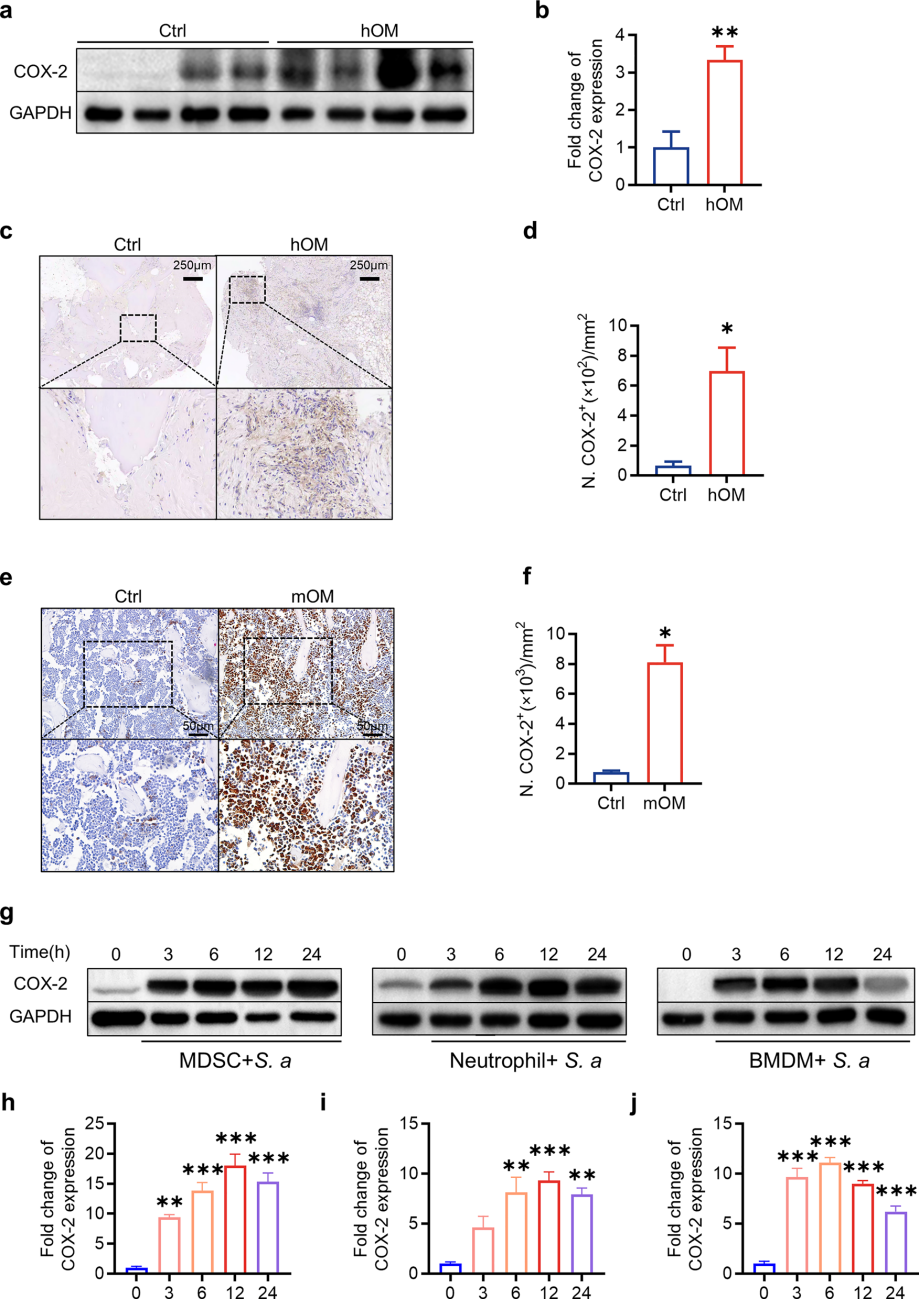
COX-2 expression was found to be upregulated in the bone marrow of osteomyelitis-affected patients and mice, as well as in immune cells infected with *S. aureus* in vitro. Typical images (**a**) of western blots and quantitative analysis (**b**) of COX-2 in the bone marrow of patients with osteomyelitis compared with controls. *n* = 4/group, ** *p* < 0.01. Typical images (**c**) and quantitative analysis (**d**) of COX-2 immunohistochemical staining in the bone marrow of patients with osteomyelitis compared with controls. Scale bars, 50 μm. *n* = 3/group, *** *p* < 0.001. Typical images (**e**) and quantitative analysis (**f**) of COX-2 immunohistochemical staining in the bone marrow of osteomyelitis mice compared with that of control mice. Scale bars, 50 μm. *n* = 3/group, * *p* < 0.05. Typical images (**g**) and quantitative analysis (**j**) of western blots for COX-2 protein levels in MDSCs (**h**), neutrophils (**i**) and BMDMs (**j**) infected with *S. aureus* (MOI = 10) for various durations (3, 6, 12 and 24 h). *n* = 3/group, ** *p* < 0.01, *** *p* < 0.001

Ultimately, our research aimed to determine whether increased COX-2 signaling contributes to the progression of *S. aureus* osteomyelitis in both humans and mice. To evaluate this, we measured COX-2 protein levels in bone marrow samples from patients with *S. aureus* osteomyelitis via western blotting and immunohistochemical staining. Our findings revealed a significant increase in COX-2 protein levels in the bone marrow of individuals with *S. aureus* osteomyelitis compared with those in the bone marrow of control participants (Fig. 7a–d). Additionally, immunohistochemical staining of femurs from hematogenous osteomyelitis mice revealed substantial upregulation of COX-2 expression in the bone marrow. To investigate the impact of *S. aureus* on COX-2 expression in immune cells, we challenged MDSCs, BMDMs and neutrophils with *S. aureus* at an MOI of 10. COX-2 expression was notably elevated at 3, 6, 12, and 24 h post-infection compared with that in the control group (0 h) in all three cell populations (Fig. 7e–g). In summary, these data demonstrate that COX-2 signaling is activated in the bone marrow of both patients and mice with *S. aureus* osteomyelitis.

## Discussion

Osteomyelitis is typically characterized by an inflammatory response that leads to bone erosion (Kavanagh et al. 2018). Chronic inflammation associated with osteomyelitis results in reduced bone strength through systemic bone loss; however, the mechanisms involved remain incompletely understood. In this study, we used IKSA to model the persistent inflammation characteristic of osteomyelitis while excluding the influence of secreted virulence factors and demonstrated that IKSA induces osteoporosis in mice. However, there were some differences in BV/TV values between experimental cohorts, which may stem from inter-vendor variability of mice. Dietary composition and nutritional protocols during developmental stages from different inter-vendor maybe contributing to the heterogeneity in baseline bone mass of mice. These foundational differences can modulate both physiological bone remodeling dynamics and the intensity of immune responses to IKSA challenge, thereby creating inter-cohort variations in osteolytic responses. Substantial evidence suggests that chronic inflammation, which suppresses the osteogenic activity of osteoblasts and enhances osteoclast-mediated bone resorption, may contribute to osteoporosis (Deng et al. 2023; Ginaldi et al. 2005). Our findings revealed that IKSA treatment resulted in a decrease in OCN^+^ cells and an increase in TRAP^+^ cells, suggesting that IKSA primarily induces bone loss by inhibiting osteogenic activity and promoting osteoclast-mediated bone resorption. Furthermore, through transcriptomic analysis of bone marrow from IKSA-treated mice, we identified several upregulated molecules, including *Ptgs2*, which are linked to the immune response and inflammation triggered by immune cells (MDSCs, neutrophils and macrophages) in response to bacterial infection. Our study demonstrated that COX-2 expression is elevated in both murine models and patients with *S. aureus* osteomyelitis. At the cellular level, both *S. aureus* infection and IKSA infection triggered COX-2 expression in bone marrow macrophages, neutrophils, and MDSCs. Inhibiting COX-2 signaling may mitigate the bone loss associated with *S. aureus* osteomyelitis. In summary, this research enhances our understanding of the role of COX-2 in regulating bone homeostasis in *S. aureus* osteomyelitis.

However, there is considerable controversy about the impact of COX-2 inhibitors, particularly celecoxib, on bone metabolism in orthopedics. Several animal and human studies have reported that delayed or impaired fracture healing is associated with the administration of COX-2 inhibitors (Gerstenfeld et al. 2007; George et al. 2020). COX-2 is essential for bone repair and has an essential function in the processes of intramembranous as well as endochondral ossification (Simon et al. 2002). It is thought that inhibiting prostaglandin production disrupts the equilibrium between bone formation and resorption during the physiological fracture healing process (Kawaguchi et al. 1995). Conversely, COX-2 inhibitors have shown antiresorptive properties and can reduce inflammatory-induced bone loss in pathophysiological processes, as demonstrated in animal studies and a few human studies (Xie et al. 2019; Gregory et al. 2006; Wanders et al. 2005; Oliveira et al. 2008), which aligns with our findings. Investigations have shown a significant reduction in the response of osteoclasts from COX-2 knockout mice to bone resorption agonists. Additionally, selective COX-2 inhibitors have been shown to block osteoclast formation in these models (Okada et al. 2000). The controversial effects of COX-2 inhibitors in regulating bone homeostasis may be attributed to differences in clinical conditions. In *S. aureus* osteomyelitis, *S. aureus* can induce excessive and persistent inflammation throughout the system, which differs significantly from the mild inflammatory response observed during fracture healing (Claes et al. 2012).

The precise mechanism by which COX-2 inhibitors exert their osteoprotective effects remains unclear. Immune system function and bone metabolism are tightly interconnected (Guerrini and Takayanagi 2014). Our study revealed that COX-2 is widely expressed in bone marrow immune cells, including MDSCs, neutrophils, and macrophages. Several studies have shown that NSAIDs influence the activity of cytokines and signaling molecules in osteoclasts, osteoblasts, and precursor cells located near the site of bone remodeling (Xie et al. 2019; Chang et al. 2023). Consistent with these reports, our data confirmed that celecoxib significantly suppressed the production of cytokines, such as RANKL, IL-1β and CXCL2, as well as the amplification of immune cells (MDSCs and neutrophils) induced by IKSA. In addition to their immunosuppressive role, MDSCs actively contribute to osteolysis by differentiating into functional osteoclasts that resorb bone (Sawant and Ponnazhagan 2013). COX-2/PGE2 signaling is crucial for the differentiation and function of MDSCs (Zhao et al. 2016). Notably, RANKL, a key molecule in osteoclast formation, is a downstream signaling molecule in the COX-2/PGE2 pathway (Pilbeam 2020). Our study demonstrated that RANKL levels were elevated in MDSCs following IKSA treatment and that celecoxib effectively reversed this increase. Furthermore, research has indicated that IL-1β promotes bone degradation while suppressing its formation in vivo (Nguyen et al. 1991). In addition to its recognized role as a chemokine, Cxcl2 contributes to the formation of osteoclasts that facilitate bone resorption (Dapunt et al. 2014). Together, these results provide a theoretical basis for the osteoprotective effects of COX-2 inhibitors in the context of *S. aureus* osteomyelitis.

Although our findings suggest that COX-2 inhibitors can improve bone loss in IKSA-treated mice, several aspects warrant further investigation. Highly virulent bacterial strains, such as *S. aureus*, trigger strong proinflammatory immune responses, in contrast to less virulent strains, such as *S. epidermidis*, which provoke more moderate immune reactions (Byrd et al. 2017). Therefore, it is important to explore whether COX-2 induction occurs similarly in response to other bacterial species. Second, different classes of NSAIDs may have varying effects on bone metabolism (Al-Waeli et al. 2021). It remains unclear whether other COX-2 inhibitors, such as meloxicam, exert osteoprotective effects comparable to those of celecoxib. Additionally, the optimal dosing regimen and the time-dependent effects of celecoxib on bone metabolism in IKSA mice have yet to be fully elucidated. Third, while our findings strongly suggest that enhanced COX-2 signaling induced by IKSA regulates both the number and inflammatory response of immune cells, the precise mechanisms by which COX-2 activation may promote osteoclastogenesis and inhibit osteoblast function require further investigation. There are also some limitations in the translation of preclinical findings to human patients. First, we employed the IKSA to simplified animal model, the complexity of human bone metabolism and the multifactorial nature of osteomyelitis in humans may not be fully replicated in mouse models. Furthermore, the immune response in humans involves a more intricate interplay of various cell types and signaling molecules, which may differ from the responses in mice.

## Conclusion

**Fig. 8:**
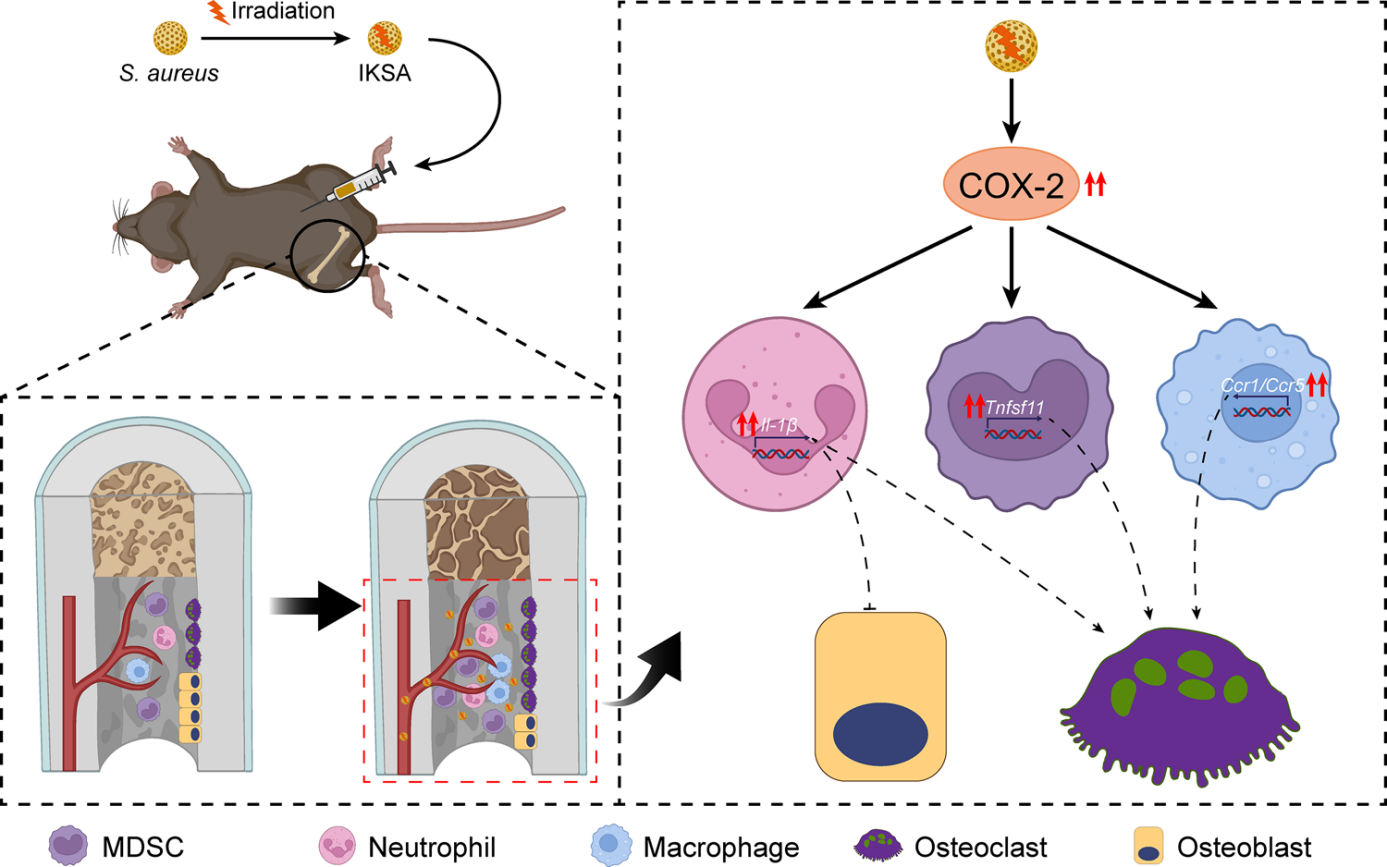
Schematic representation of bone loss induced by IKSA. IKSA treatment led to the amplification of immune cells, including MDSCs, neutrophils, and macrophages, in the bone marrow, concurrently increasing COX-2 expression in these immune populations. The increased COX-2 levels may, in turn, drive the upregulation of Tnfsf11 in MDSCs, Il1b in neutrophils, and Ccr1/Ccr5 in macrophages. This signaling cascade is likely to contribute to a reduction in bone mass by promoting osteoclast activity while inhibiting osteogenesis

In conclusion, our research indicates that COX-2 may be a key molecular target mediating bone loss in *S. aureus*-induced osteomyelitis. Our data show that the COX-2 inhibitor celecoxib may prevent bone loss associated with *S. aureus* osteomyelitis by regulating the number and inflammatory response of bone marrow immune cells (Fig. 8). As a widely prescribed medication for pain and inflammation in osteomyelitis patients, celecoxib may offer osteoprotective benefits beyond its anti-inflammatory and pain-relieving properties. This study provides substantial experimental evidence supporting the potential of COX-2-targeted therapies as novel approaches for treating bone loss in patients with osteomyelitis. Based on the current findings and the limitations of this study, future research directions should focus on the following prioritized objectives. Randomized controlled trials investigating the role of COX-2 in bone metabolism among clinical osteomyelitis populations should be conducted. These trials should implement standardized inclusion criteria incorporating disease stage classifications, and evaluate the safety and efficacy of COX-2 inhibitors in human subjects. Additionally, the optimal dose and type of specific COX-2 inhibitor for the treatment of osteomyelitis should be determined. In brief, future research should focus on further investigating COX-2-targeted therapies to optimize their clinical benefit for human.

## Supplementary Information

Additional file 1. Figure S1. Gating strategy. Figures showing the gating strategy used to detect MDSC, neutrophils and macrophages.

Additional file 2. Figure S2. IKSA induced cortical bone loss by suppressing bone formation and activating bone resorption in endocortical envelope.Typical images and quantitative analysis of OCN immunohistochemical staining in the endocortical envelope of femurs from IKSA-treated and control mice. n = 3/group, ***p < 0.001. Scale bars, 100 μm. Red arrows indicate OCN^+^ cells.Representative TRAP staining images and quantification in the endocortical envelope of femurs from IKSA-treated and control mice. Scale bars, 100 μm. n = 3/group, **p < 0.01.

Additional file 3. Figure S3. The effect of celecoxib to Bone formation and resorption in endocortical envelope of bone.Typical images and quantitative analysis of OCN immunohistochemical staining in the endocortical envelope of femurs from vehicle control mice and IKSA-treated mice with intragastric administration of vehicle or celecoxib. n = 3/group, **p < 0.01. Red arrows indicate OCN^+^ cells. Scale bars, 100 μm.Typical TRAP staining images and quantitative analysis in the endocortical envelope of femurs from vehicle control mice and IKSA-treated mice with intragastric administration of vehicle or celecoxib. Scale bars, 100 μm. n = 3/group, **p < 0.01.

## Data Availability

The data that support the findings of this study are available from the corresponding author upon reasonable request.
