# Supplementary figures and images for: COX-2 inhibition as a therapeutic strategy for bone loss in Staphylococcus aureus osteomyelitis
Source: Mol Med. 2025 May 7;31:177. doi: 10.1186/s10020-025-01202-9 (PMC12057237; doi:10.1186/s10020-025-01202-9)

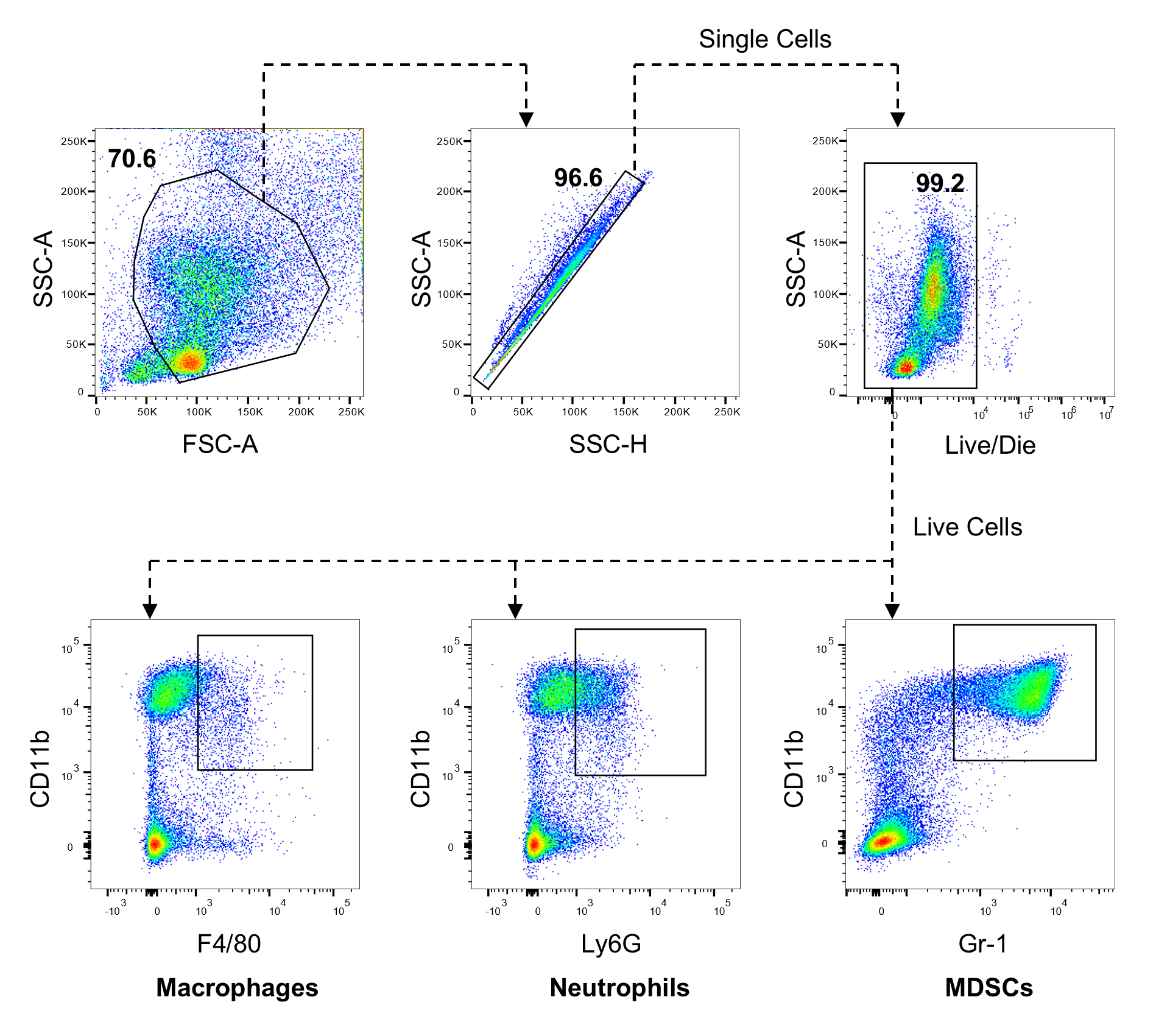

Supplement: Supplementary file 1 — Additional file 1. Figure S1. Gating strategy. Figures showing the gating strategy used to detect MDSC, neutrophils and macrophages. [file 10020_2025_1202_MOESM1_ESM.tif]

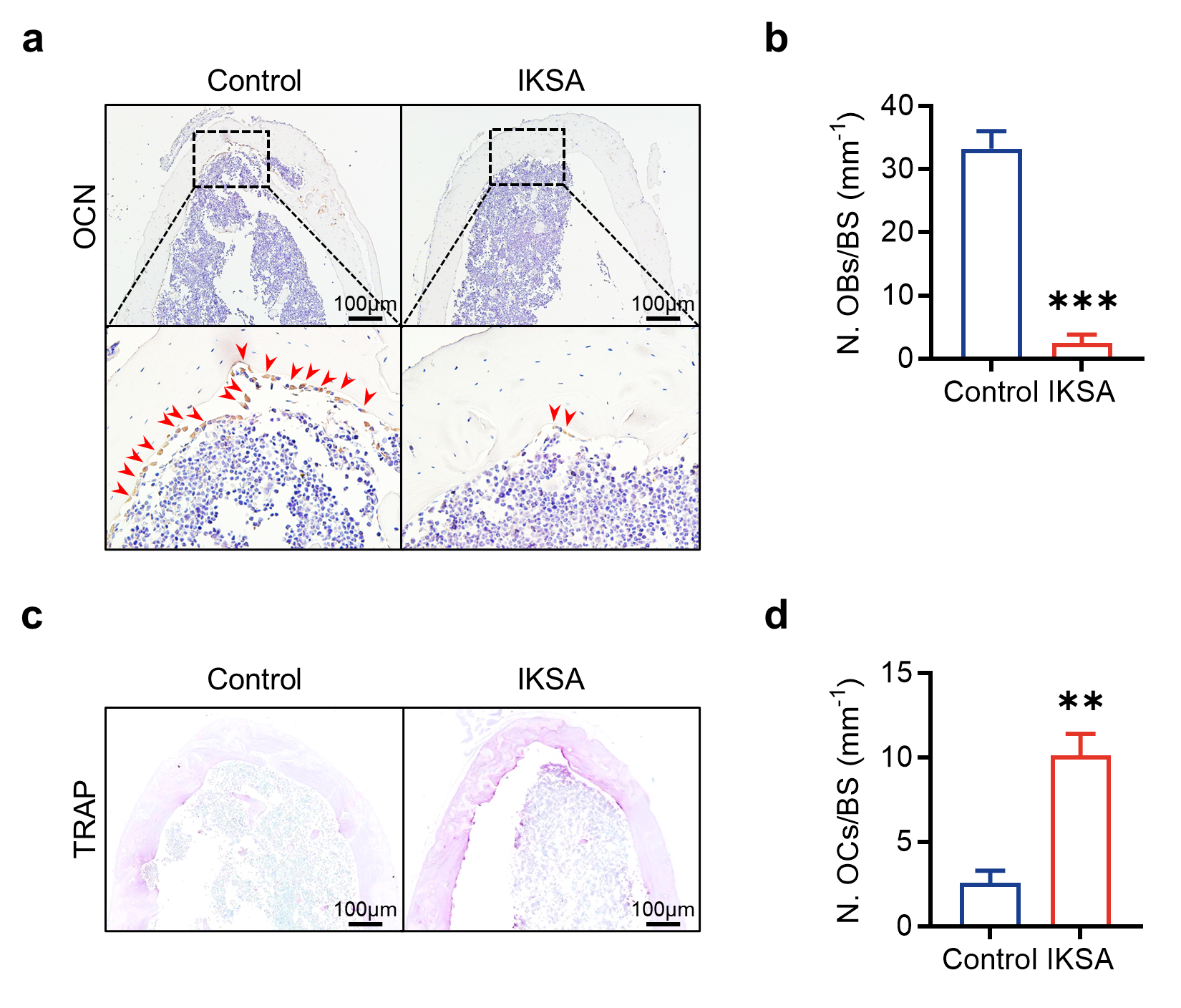

Supplement: Supplementary file 2 — Additional file 2. Figure S2. IKSA induced cortical bone loss by suppressing bone formation and activating bone resorption in endocortical envelope.Typical images and quantitative analysis of OCN immunohistochemical staining in the endocortical envelope of femurs from IKSA-treated and control mice. n = 3/group, ***p < 0.001. Scale bars, 100 μm. Red arrows indicate OCN+ cells.Representative TRAP staining images and quantification in the endocortical envelope of femurs from IKSA-treated and control mice. Scale bars, 100 μm. n = 3/group, **p < 0.01. [file 10020_2025_1202_MOESM2_ESM.tif]

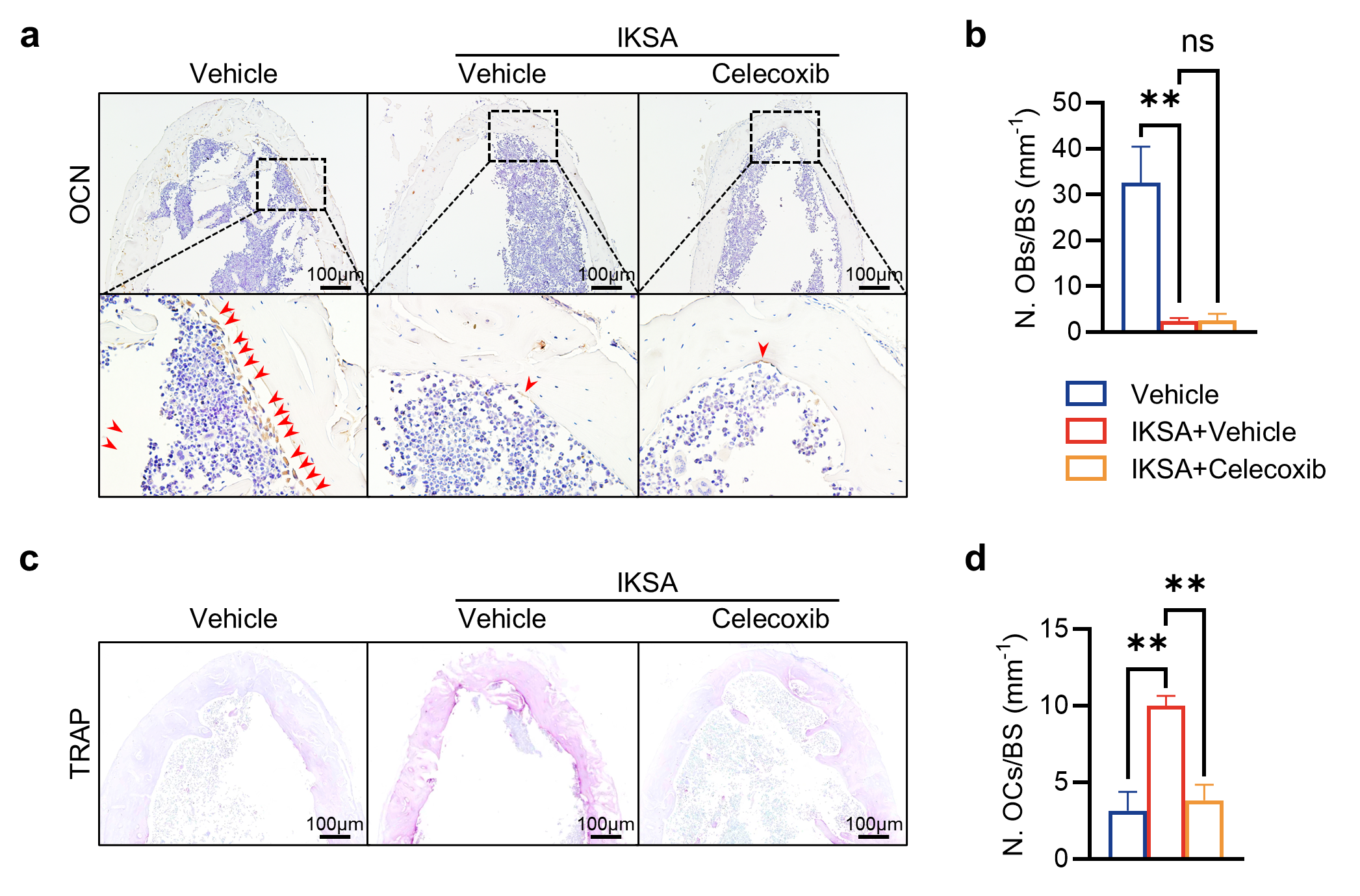

Supplement: Supplementary file 3 — Additional file 3. Figure S3. The effect of celecoxib to Bone formation and resorption in endocortical envelope of bone.Typical images and quantitative analysis of OCN immunohistochemical staining in the endocortical envelope of femurs from vehicle control mice and IKSA-treated mice with intragastric administration of vehicle or celecoxib. n = 3/group, **p < 0.01. Red arrows indicate OCN+ cells. Scale bars, 100 μm.Typical TRAP staining images and quantitative analysis in the endocortical envelope of femurs from vehicle control mice and IKSA-treated mice with intragastric administration of vehicle or celecoxib. Scale bars, 100 μm. n = 3/group, **p < 0.01. [file 10020_2025_1202_MOESM3_ESM.tif]
